# The significance of gut microbiota in the etiology of autoimmune hepatitis: a narrative review

**DOI:** 10.3389/fcimb.2024.1337223

**Published:** 2024-02-09

**Authors:** Chen Sun, Dongzi Zhu, Qi Zhu, Zeping He, Yichao Lou, Desheng Chen

**Affiliations:** ^1^ Clinical Research Center, Shanghai General Hospital, Shanghai Jiao Tong University School of Medicine, Shanghai, China; ^2^ Department of General Surgery, Shanghai Ninth People’s Hospital, Shanghai Jiao Tong University School of Medicine, Shanghai, China; ^3^ Department of General Surgery, Shanghai General Hospital, Shanghai Jiao Tong University School of Medicine, Shanghai, China

**Keywords:** autoimmune hepatitis, intestinal microbiota, metabolites, autoimmunity, intestinal barrier

## Abstract

Autoimmune hepatitis (AIH) is a chronic inflammatory disease of the liver that is mediated by autoimmunity and has complex pathogenesis. Its prevalence has increased globally. Since the liver is the first organ to be exposed to harmful substances, such as gut-derived intestinal microbiota and its metabolites, gut health is closely related to liver health, and the “liver-gut axis” allows abnormalities in the gut microbiota to influence the development of liver-related diseases such as AIH. Changes in the composition of the intestinal microbiota and its resultant disruption of the intestinal barrier and microbial transport are involved in multiple ways in the disruption of immune homeostasis and inflammation, thereby influencing the development of AIH. In terms of the mechanisms involved in immune, the gut microbiota or its metabolites, which is decreased in secondary bile acids, short-chain fatty acids (SCFAs), and polyamines, and increased in lipopolysaccharide (LPS), branched-chain amino acids (BCAA), tryptophan metabolite, amino acid, and bile acid, can disrupt immune homeostasis by activating various immune cells and immune-related signaling pathways, resulting in aberrant activation of the immune system. Clarifying this mechanism has significant clinical implications for the treatment of AIH with drugs that target intestinal microbiota and related signaling pathways. Therefore, this narrative review summarizes the progress in exploring the involvement of gut microbiota in the pathogenesis of AIH, with the aim of helping to improve the precise targeting of therapeutic treatments against AIH for the benefit of clinical AIH treatment.

## 1 Introduction

Autoimmune hepatitis (AIH) is an autoimmune disease that has a high risk of progressing cirrhosis and end-stage liver failure if not promptly diagnosed and treated (Muratori et al., 2023). Although the precise cause of AIH is still unknown, genetic factors, environmental factors, and autoimmune reactions are closely linked to the etiology of AIH (Manns et al., 2015; Floreani et al., 2018).

Advances in genome sequencing technology, bioinformatics, and culturomics have led to a further understanding of the characterization and function of the gut microbiota (Macori and Fanning, 2023; Shen et al., 2023). The gut microbiota is composed of bacteria, archaea, eukaryotes, viruses, and parasites (Weiner et al., 2023). The interplay amidst the gut microbiota and the host’s intestinal milieu assumes a pivotal function in the realms of digestion, metabolism, immunity, inflammation, and afflictions (Macori and Fanning, 2023). Since the liver receives the majority of its blood supply from the intestine, alterations in the intestine’s microbiota and metabolites can have a significant impact on the progression of liver pathology. In recent years, a growing body of evidence suggests that the gut microbiota may affect the progression of liver disease via the gut/microbiome-liver axis (Albillos et al., 2020; Hov and Karlsen, 2023). The gut microbiota dysbiosis plays an important role in autoimmune-mediated diseases including AIH (Liu et al., 2021a). Several studies have emphasized the importance of “gut-liver crosstalk” in the pathogenesis of AIH (Liang et al., 2021; Wang et al., 2021). Microorganisms have the ability to impact intestinal immune cells by presenting diverse molecular structures that can activate innate immune sensors and/or adaptive immune receptors. Segmented filamentous bacteria (SFB), which are commensal bacteria derived from Taconic mice, exhibit a specific colonization pattern in the ileum. They have the ability to induce the differentiation of T helper (Th) 17 cells and activate type 3 innate lymphoid cells (ILC3s) (Park et al., 2023). In addition, *Clostridia*, a prevalent class of commensal microorganisms, are capable of triggering the development of regulatory T (Treg) cells within the colon by instilling transforming growth factor-β1 (TGF-β1) and indoleamine-2,3 dioxygenase (IDO) in the immune microenvironment. These Treg cells play a crucial role in suppressing inflammatory and allergic responses (Ramanan et al., 2023). However, the specific mechanisms of the gut microbiota and its metabolites implicated in the development of AIH have not been fully elucidated, and there are an increasing number of clinical and basic studies focusing on the microbiota for the treatment of AIH. Thus, a narrative review of the relationship between the microbiota and its metabolites and the development of AIH is urgently required to investigate the molecular mechanisms and provide new insights into the clinical management of AIH.

This narrative review will describe the relationship between the gut microbiota and its metabolites and the pathogenesis of AIH, and summarize the specific mechanisms of the gut microbiota and its metabolites involved in the process of the development of AIH from multiple perspectives. It contributes to the clinical therapeutic challenges, with the aim of providing new targets and new ideas for the future treatment of AIH.

## 2 Involvement of gut microbiota in AIH development

Patients with AIH have intestinal microflora that exhibit symbiosis. Their intestinal microflora is characterized by decreased bacterial diversity and altered bacterial relative abundance compared to that of healthy individuals (Cheng et al., 2022). The altered abundance of different gut microbiota in AIH patients versus healthy individuals is shown in **Table 1**. AIH is associated with a substantial decrease in the diversity of the gut microbiome and an increase in the relative abundance of aerobic or partially anaerobic microbes (Cheng et al., 2022). A study revealed a striking disparity between specific pathogen-free (SPF) mice and germ-free (GF) mice in terms of their susceptibility to Concanavalin A (ConA)-induced liver injury. The research findings demonstrated that GF mice exhibited resistance to such liver injury, in sharp contrast to SPF mice (Wei et al., 2016). However, the trend of *Proteobacteria, Firmicutes*, and *Bacteroidetes* in various research is still controversial and requires more authoritative studies and a larger number of patients and normal subjects (Elsherbiny et al., 2020; Wei et al., 2020). To understand the composition and relative abundance of intestinal microbiota, the detection of 16S rRNA gene sequencing has some clinical significance in deducing the pathogenesis of AIH through the characterization of intestinal microbiota. However, the results of different studies vary considerably. Manfredo Vieira et al. have confirmed that one of the culprits of systemic lupus erythematosus (SLE) and AIH is *E. gallinarum*, which has been discovered in the livers of patients with SLE and AIH (Manfredo Vieira et al., 2018). However, Wei et al. found no difference in the relative abundance of *E. gallinarum* in 28 AIH patients and 34 healthy controls (Wei et al., 2020). A study found significantly lower proportions of *Bifidobacterium* and *Lactobacillus* in AIH by comparing *Bifidobacterium, Lactobacillus, Escherichia coli*, and *Enterococcus* in 24 patients with AIH and 8 healthy individuals (Lin et al., 2015). Similarly, another study involving 15 AIH patients and 10 healthy individuals as controls, whose fecal samples were sequenced by 16S rRNA gene sequencing, found that AIH patients had a greater abundance of *Bacteroides, Clostridium, Lactobacillus, Bifidobacterium*, and *Eubacterium* (Elsherbiny et al., 2020). *Veillonella* is associated with elevated levels of aspartate aminotransferase (AST) and liver inflammation and is closely associated with AIH in *Veillonella*, *Streptococcus, Klebsiella*, and many other genera (Wei et al., 2020). Therefore, the diversity and proportional changes in the microbiota may have clinical significance for the early detection and differentiation of AIH patients from the healthy population. Through modeling and cohort validation, the combination of *Veillonella, Lactobacillus, Oscillospira*, and *Clostridium* was effective in differentiating patients with AIH from normal healthy individuals, with an area under the an area under curve (AUC) for the probability of disease of 78% (95% CI 0.71 to 0.84), with *Veillonella* correlating with the severity of AIH *Veillonella* was associated with AIH severity. These findings suggested a superior predictive capability, which could enhance patient evaluation and aid in making clinical decisions regarding management (Liwinski et al., 2020; Wei et al., 2020). Additionally, *Roseburia, Bacteroides, Lachnospiraceae, Veillonella*, and *Ruminococcaceae* discriminated between AIH patients and healthy individuals with AUC that could reach 83.25% (95% CI 0.76 to 0.91) (Lou et al., 2020). As several factors could affect the composition and proportion of the gut microbiota, and as there is variation between studies regarding the gut microbiota of AIH patients, the study requires more rigorous and extended follow-up cohort studies.

**Table 1:** Changes in the relative amount of gut microbiota in AIH patients compared to healthy individuals.

Microbiota	Relative abundance	Reference
*Veillonella*	Increase	(Wei et al., 2020)
*Streptococcus*	Increase	
*Klebsiella*	Increase	
*Lactobacillus*	Increase	
*Synergistetes*	Decrease	(Cheng et al., 2022)
*Lentisphaerae*	Decrease	
*Clostridiales*	Decrease	(Wei et al., 2020)
*Coprococcus*	Decrease	
*RF39*	Decrease	
*Ruminococcaceae*	Decrease	
*Rikenellaceae*	Decrease	
*Oscillospira*	Decrease	
*Parabacteroides*	Decrease	
*E. gallinarum*	Increase	(Manfredo Vieira et al., 2018)
*E. gallinarum*	NS	(Wei et al., 2020)
*Bifidobacterium*	Decrease	(Lin et al., 2015)
*Lactobacillus*	Decrease	
*Bacteroides*	Increase	(Elsherbiny et al., 2020)
*Clostridium*	Increase	
*Lactobacillus*	Increase	
*Bifidobacterium*	Increase	
*Eubacterium*	Increase	
*Lachnospiraceae*	Decrease	(Lou et al., 2020)
*Veillonella*	Increase	
*Bacteroides*	Increase	
*Roseburia*	Increase	
*Ruminococcaceae*	Increase	

In order to investigate the impact of antibiotics and microbiota on children diagnosed with AIH/primary sclerosing cholangitis (PSC) overlap syndrome or PSC alone, a clinical study was conducted to assess alterations in the gut microbiota [NCT03069976]. This study aimed to understand the role of microbiota in the pathogenesis of AIH/PSC overlap syndrome and PSC in children. The majority of patients with AIH have an insidious onset of disease, while a small number have an acute onset and lack due to the lack of effective interventions, often progressing to cirrhosis, hepatic failure, and other manifestations of hepatic dysfunction. Fecal microbiota transplantation (FMT) is a well-established therapeutic approach to stably alter the gut microbiome and has been shown to be safe and effective in several disease states resulting from intestinal dysbiosis. Two additional clinical studies evaluated whether alteration of the gut microbiome by FMT could ameliorate hepatic dysfunction and inhibit malignant progression of liver function by alleviating intestinal dysbiosis and immune dysfunction [NCT02862249, NCT03014505].

**Figure 1:**
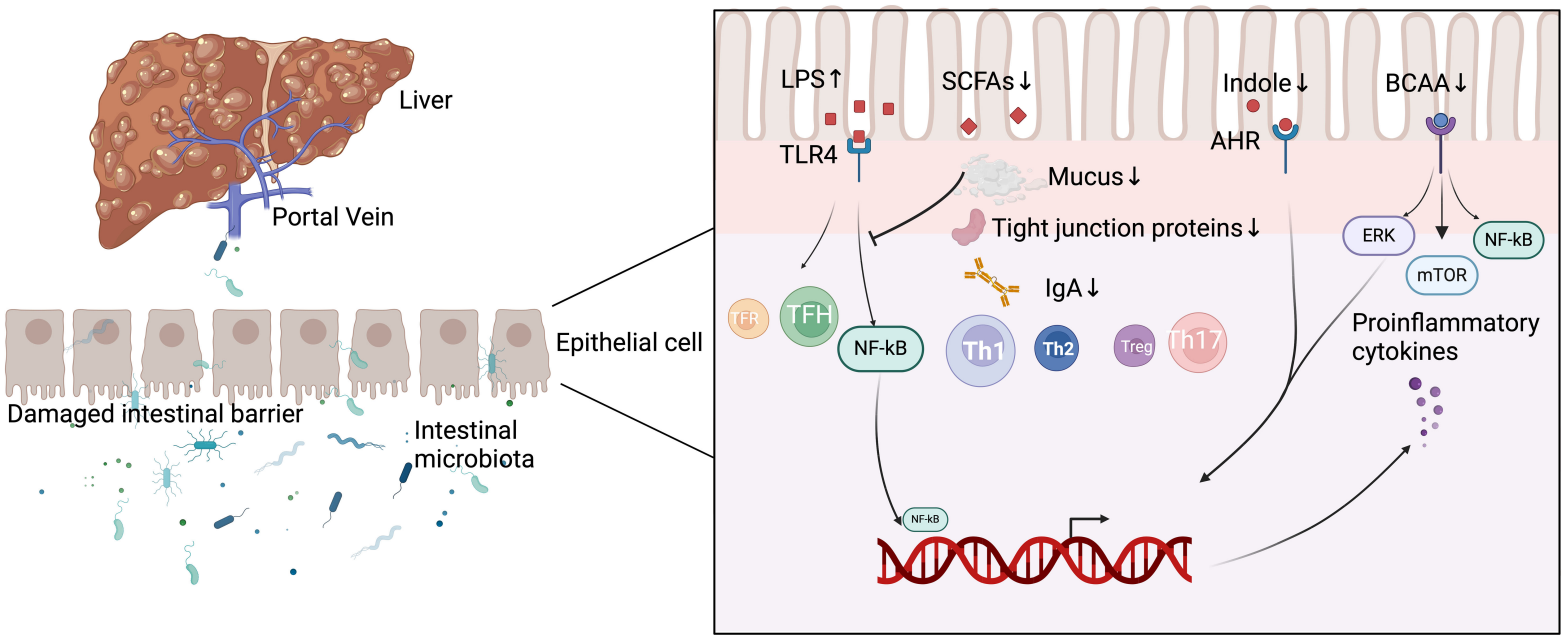
Mechanisms of intestinal microbiota involved in the development of AIH. LPS, lipopolysaccharide; SCFAs, short-chain fatty acids; AHR, aryl hydrocarbon receptor; BCAA, branched-chain amino acids; TFR, follicular regulatory T; TFH, follicular helper T; Th 1, T helper 1; Th 2, T helper 2; Th 17, T helper 17; TLR4, toll-like receptor 4; NF-kB, nuclear factor-kappa B; ERK, extracellular regulated protein kinases; mTOR, mammalian target of rapamycin; IgA, Immunoglobulin A.

Yuksel et al. developed human leukocyte antigen DR3 nonobese-diabetic (HLA-DR3 NOD) mice as a ‘humanized’ mouse model of AIH by immunizing human HLA-DR3 transgenic NOD mice with cytochrome P4502D6 protein/formiminotransferase cyclodeaminase (CYP2D6/FTCD). They discovered that the composition of the intestinal microbiota in HLA-DR3 mice with liver injury was substantially different from that of wild-type nonobese-diabetic (WT NOD) mice with liver injury. This demonstrates that interactions between gut microbiota and the mucosal immune system may be one of the primary causes of AIH, but its definitive mechanism in the development of AIH still needs to be investigated in additional human and animal models (Yuksel et al., 2015). In recent years, as people have become more aware of AIH, an increasing number of studies have demonstrated that gut microbiota-induced processes, such as gut barrier disruption, gut microbiota migration, altered gut microbial metabolism, and disruption of immune homeostasis, play crucial roles in the development and progression of AIH (Wang et al., 2023, Li and Kang, Terziroli Beretta-Piccoli et al., 2022). **Figure 1** summarizes the current understanding of the mechanisms by which intestinal microbiota contributes to AIH.

### 2.1 Disruption of the intestinal barrier and intestinal microbiota translocation

The intestinal barrier, which consists of microbial, chemical, mechanical, and immune barriers, is an innate barrier that maintains a tranquil intestinal environment and prevents harmful substances, such as bacteria and endotoxins, from leaving the intestine and entering other tissues and organs of the body. Functional intestinal barrier integrity is required to prevent bacterial transfer (Kronsten et al., 2022). The integrity of the intestinal barrier is closely related to intestinal villi, tight junctions between cells, normal intestinal microbiota, and related cytokine proteins (Lin et al., 2023). In addition, the tightness and integrity of transmembrane and cytoplasmic protein junctions are also critical for maintaining the normal function of the intestinal barrier (Alizadeh et al., 2022).

#### 2.1.1 Gut microbial

The intestinal tract hosts many species of commensal bacteria, which are constantly stimulated by foreign dietary and environmental antigens and maintain a dynamic equilibrium while contributing to the body’s normal physiological functions (Airola et al., 2023). Under normal physiological conditions, the intestinal microbiota plays a key role in maintaining the normal structure of the gut, promoting the integrity of the intestinal barrier, and modulating the mucosal immune response by maintaining intercellular connections and promoting epithelial repair (Gupta and Dey, 2023; Iancu et al., 2023). A study evaluates the correlation of changes in gut permeability, bacterial transit, and gut microbiome with AIH progression in patients with AIH. According to this study, AIH was associated with disturbed intestinal microbiota and damaged intestinal barrier (Lin et al., 2015). Dysbiosis of the intestinal microbiota through a variety of mechanisms leading to impaired intestinal villi or insufficient tightness and integrity of connexins may affect the integrity of the intestinal barrier, thereby facilitating bacterial translocation and affecting the normal physiological functioning of the liver. Normal duodenal villi have a finger-like or leaf-like structure. Compared to the typical structures observed in healthy individuals, study has revealed modified intestinal tight junctions and inflammatory infiltrates within the lamina propria of AIH patients. These modifications result in damage to the intestinal mucosa and elevated levels of endotoxins in the bloodstream, thereby triggering the immune tolerance impairment mechanism and causing liver injury (Kharrazian et al., 2023; Zhang et al., 2023b; Zhu et al., 2023). Under normal physiological conditions, anaerobic/aerobic bacteria in the intestinal microbiota maintain a dynamic balance and participate in physiological processes, and the disruption of this balance also can lead to intestinal barrier damage and bacterial translocation across the intestinal barrier to other organs, resulting in inflammation, immune disorders, and various other adverse effects (Lin et al., 2015).

#### 2.1.2 Metabolites of gut microbial

Different metabolic patterns are utilized by intestinal microbiota to conduct complex and active metabolic reactions in the intestinal tract. Using the undigested food of the host and the secretions of intestinal epithelial cells as substrates, they generate a large number of metabolites that are either beneficial or detrimental to the human body. In addition to changes in intestinal microflora, changes in the composition and abundance of its metabolites play a significant role in maintaining the integrity and tensile strength of the intestinal barrier. These metabolites also impact the intestinal barrier integrity of the host in various ways, either directly or indirectly, leading to an intestinal inflammatory response and the development of liver-related autoimmune diseases such as AIH.

In AIH disease models, dysbiosis induces metabolic alterations in the gut microbiota, which in turn disrupt gut barrier integrity and immune homeostasis (Cheng et al., 2022). Changes in gut microbiota metabolites in patients with AIH include decreased concentrations of short-chain fatty acids (SCFAs), tryptophan metabolite, secondary bile acids, and polyamines, and increased concentrations of branched-chain amino acids (BCAA) (Elsherbiny et al., 2020; Kayama et al., 2020; Liwinski et al., 2020; Lou et al., 2020; Wei et al., 2020). Among these, SCFAs (Liwinski et al., 2020; Lou et al., 2020), lipopolysaccharide (LPS) (Wei et al., 2020), and tryptophan metabolite (Kayama et al., 2020) are significant for maintaining the integrity of the intestinal barrier.

The microbiota in the intestine digests indigestible polysaccharides into SCFAs to provide energy to the cells. As an important metabolite of microorganisms, SCFAs, consisting of propionic acids, acetic acids, and butyric acids, are essential for the maintenance of intestinal integrity, not only regulating intestinal pH, increasing mucus production, up-regulating the expression of tight junction proteins, providing fuel for epithelial cells, and affecting mucosal immune function (Blaak et al., 2020). The reduction in SCFAs in AIH patients may be associated with disruption of the intestinal barrier and subsequent progression of AIH (Cheng et al., 2022). Butyric acid, the most important component of SCFAs, can induce mucin expression by stimulating intestinal epithelial cells (IECs), causing changes in bacterial adherence and intestinal barrier tightness (Liu et al., 2022), which in turn affects the development of AIH.

In addition to the direct effects of SCFAs on the intestinal barrier described previously, it has been demonstrated that SCFAs can also function as beneficial microbiota metabolites in the liver-gut axis to inhibit LPS-induced inflammatory responses (Gao et al., 2023; Yan et al., 2023). LPS, or endotoxin, is a component found in the outer membrane of the majority of Gram-negative bacteria. It plays a vital role in the gut-liver axis by triggering the activation of immune cells, such as Kupffer cells, and leading to the release of pro-inflammatory cytokines, among other effects (Babu and Mohanty, 2023; Brock and Cooper, 2023). The abnormal metabolism of intestinal microflora in AIH patients leads to an increase in the ability to synthesize LPS and the relative abundance of LPS in the intestine (Wei et al., 2020). Additionally, *Veillonella*, which is strongly associated with AIH, also produces LPS (Fan et al., 2023). The intestinal bacterial membrane component LPS can activate toll-like receptor 4 (TLR4), resulting in reduced expression of intestinal barrier tight junction proteins and impaired intestinal barrier (Liu et al., 2021b). Similarly, the pathway by which LPS leads to gut barrier disruption also includes activation of the downstream nuclear factor kappa B (NF-κB) signaling pathway (Liu et al., 2021b). Zonulin, a biomarker associated with the integrity of barriers and playing a vital role in the regulation of intercellular tight junction function, triggers the phosphorylation of zonula occludens proteins, resulting in the disassembly of tight junctions and an elevation in intestinal permeability. Tulkens et al. found a significant positive correlation between bacterial extracellular vesicles (EV)-associated LPS and the amount of zonulin in plasma by analyzing plasma from 49 subjects (Spearman’s r=0.4241, p=2.45×10^-2^) (Tulkens et al., 2020). Therefore, increased abundance of LPS due to aberrant metabolism of intestinal microorganisms leads to increased zonulin content, which in turn leads to increased intestinal permeability, resulting in adverse effects such as intestinal inflammation and bacterial translocation.

Tryptophan that enters the intestine is broken down by tryptophanase to indole, which can enhance intestinal barrier function by activating the pregnane X receptor (PXR) and aryl hydrocarbon receptor (AHR). Consequently, tryptophan deficiency also affects intestinal barrier integrity (Kayama et al., 2020). Among them, AHR is present in various immune cells and endothelial cells, and activation of AHR via indole and tryptamine contributes to the production of various immune-associated cytokines, such as interleukin-10 (IL-10), interleukin-17 (IL-17), interleukin-22 (IL-22), thereby regulating intestinal immune homeostasis. Furthermore, activation of PXR regulates intestinal barrier mucosal integrity mediated by TLR4 (Ye et al., 2022). Indole acts as a ligand for the AHR, and the secretion of IL-22 upon AHR activation also plays an important role in maintaining normal epithelial barrier function (Ye et al., 2022). IL-22-producing cells consist of Th1 cells, Th 22 cells, CD8^+^T cells, Th 17 cells, and other lymphocytes, and the efficient production of IL-22 is closely related to the activation of AHR (Mar et al., 2023). Mucus serves as the first line of defense against the invasion of microorganisms and pathogens, protecting intestinal health. The regulation of epithelial homeostasis includes the regulation of epithelial cell proliferation and permeability, as well as the regulation of the production of mucus, complement, and antimicrobial proteins (AMPs) (Keir et al., 2020). IL-22 modulates the growth and permeability of epithelial cells and influences the production of mucin, complement, and AMPs to maintain epithelial homeostasis. In addition, IL-22 can increase the amount of mucus in mucosal tissues by increasing the number of cuprocytes. Consequently, the expression of mucins produced by cuprocytes and activation of Signal Transducer and Activator of Transcription 3 (STAT3)-dependent signaling induces the expression of mucin-associated genes in mucosal epithelial cells, etc. Therefore, the key role played by tryptophan metabolite in the development of AIH should not be overlooked.

#### 2.1.3 Other protein components

Several important small molecules are also involved in the alteration of the intestinal barrier. Polymeric immunoglobulin receptor (pIgR) is a single transmembrane protein and a key regulator of AIH. Its extracellular secretory components can be released in the form of secretory immunoglobulin A (sIgA), but the deficiency of pIgR will reduce the intestinal sIgA level and increase the intestinal barrier dysfunction, leading to more severe intestinal microbiota imbalance, increased bacterial migration, and accelerated development of AIH (Lin et al., 2023). sIgA possesses antimicrobial properties that inhibit aberrant immune responses triggered by intestinal microorganisms and prevent autoimmune reactions (Lin et al., 2023). In addition, intestinal epithelial pIgR is closely associated with intestinal barrier destruction and subsequent liver injury in experimental AIH mice (Lin et al., 2023).

Under healthy conditions, only a limited number of bacteria and their metabolites can reach the liver and be eliminated by the immune system of the liver. Under pathological conditions, however, disruption of the microbiota causes an increase in intestinal permeability, which results in the transfer of bacteria from the intestines to the liver via the circulation, thereby triggering liver inflammation and fibrosis (Bruneau et al., 2021; Bragazzi et al., 2023; Jiang et al., 2023). Consequently, the degradation of the intestinal barrier and the increase in intestinal permeability caused by intestinal microorganisms, their metabolites, and associated proteins are also essential components of the pathogenesis of AIH.

### 2.2 Destruction of immune homeostasis

The intestinal barrier is also essential for preventing the translocation of intestinal microbiota and maintaining intestinal immune homeostasis (Chopyk and Grakoui, 2020). AIH’s immune-related pathogenesis is more intricate and inadequately articulated. A significant contributor to the development of AIH is the abnormal activation of intestinal microflora and its metabolites on the innate immune system. It is well known that the target antigen of the majority of AIH is an unidentified autoantigen or a foreign antigen similar to an autoantigen. Intestinal microorganisms and their metabolites as antigens are capable of interacting with mucosal immune cells and influencing the body’s immune response, thereby influencing the development of AIH via immune pathways. Excessive production of gut-derived toxic metabolites may disrupt normal liver physiology via abnormal activation of the innate immune system and activation of signaling pathways and related receptors associated with hepatic inflammation, leading to hepatic inflammation and hepatic autoimmune diseases such as AIH.

#### 2.2.1 Gut microbial metabolites

The gut microbiota and its metabolites can modulate immune responses in numerous ways to either maintain intestinal homeostasis or induce intestinal inflammation. The gastrointestinal tract is essential for preventing gut microbiota exposure and maintaining immune homeostasis in the host (Chopyk and Grakoui, 2020; Bragazzi et al., 2023). Numerous factors influence the constitution and abundance of intestinal microflora, so its composition is not static. Alterations in gastrointestinal microbiota metabolism result in changes in metabolite concentrations (decrease in secondary bile acids, SCFAs, and polyamines, and increase in BCAA), which affect immune homeostasis in various ways and promote the development of AIH (Elsherbiny et al., 2020; Liwinski et al., 2020; Lou et al., 2020; Wei et al., 2020).

Bile acids are important metabolites of the microbial community that can directly or indirectly affect the microbial community by activating the innate immune system (Guo et al., 2022). Bile acid metabolism can be affected by *Bacteroides*, *Clostridium*, *Lactobacillus*, *Bifidobacterium*, and *Eubacterium*, which are enriched in AIH patients (Elsherbiny et al., 2020). In a Con A-induced mouse model of hepatitis, it was discovered that a decrease in the relative amount of *Clostridium* in the intestinal tract and a concomitant reduction in the number of secondary bile acids secreted by *Clostridium* attenuated liver injury by inhibiting its activation of the G protein-coupled bile acid receptor 1 (GPBAR1) on the surface of natural killer T (NKT) cells and suppressing their polarization to NKT 10 cells and the secretion of IL-10 (Biagioli et al., 2019). Since SCFAs can affect mucosal immune function, their composition and relative abundance are also closely associated with health status (Ríos-Covián et al., 2016; Blaak et al., 2020). SCFAs have multiple effects on T cell activation and their immune effector functions. By inhibiting histone deacetylases (HDACs), butyrate and propionate may also play a significant role in inducing the differentiation and anti-inflammatory properties of T regulatory cells (Xu et al., 2022; Blake et al., 2023; Khantakova and Sennikov, 2023; Yang et al., 2023). Moreover, polyamines could facilitate the differentiation and maturation of intestinal immune cells, whereas metabolic disorders in the intestinal microbiota led to altered arginine metabolism and decreased serum polyamine levels, thereby altering the normal intestinal immune response (Wei et al., 2020; Cheng et al., 2022). Meanwhile, BCAA including valine, leucine, and isoleucine, are also involved in the intestinal immune process, which can be involved in the up-regulation of innate and adaptive immune responses through the activation of various signaling pathways [isoleucine: G-Protein Coupled Receptor, extracellular regulated protein kinases (ERK) signaling pathways; leucine: mammalian target of rapamycin (mTOR); valine: NF-kB]. In addition, the metabolism of BCAA is upregulated in AIH patients relative to normal controls. Cirrhosis is a clinical manifestation associated with AIH, and its increased levels of relevant proteins are linked to two metabolic pathways, namely map00290 (biosynthesis of valine, leucine, and isoleucine) and map00770 (biosynthesis of pantothenate and coenzyme A) (Elsherbiny et al., 2020).

#### 2.2.2 Gut microbes and their metabolite transport

Various factors, including intestinal microorganisms, degrade the intestinal barrier function, and intestinal permeability increases, intestinal bacteria are more likely to transfer to external organs, and the innate immune system is stimulated, resulting in liver inflammation and liver damage. The toll-like receptor (TLR) pathway plays a crucial function in the activation of the host immune response by intestinal microorganisms (Kayama et al., 2020). Gut microbes and their metabolite transport, including LPS and bacterial DNA, can effectively activate the immune response through the activation of TLR, which in turn causes liver injury, and the degree of increase in the plasma level of LPS in patients with AIH is significantly correlated with the severity of AIH (Papadakos et al., 2023; Verma et al., 2023).

The immune system also uses the secretion of antimicrobial peptides and Immunoglobulin A (IgA) to safeguard the intestinal barrier and maintain immune homeostasis (Chopyk and Grakoui, 2020). IgA is a significant component of the intestinal mucosa and plays a key role in maintaining intestinal immune homeostasis by regulating intestinal microbiota translocation and growth and even inhibiting or killing bacteria (Hapfelmeier et al., 2010; Gutzeit et al., 2014; Nakajima et al., 2018). IgA-mediated transport by pIgR is involved in mucosal immunity and plays an important role in regulating the development of AIH (Kaetzel, 2005; Lin et al., 2023). IgA binds to various bacteria to induce distinct IgA-type immune responses. Under normal physiological conditions, the intestinal tract of healthy individuals produces IgA to maintain intestinal homeostasis. Consequently, IgA and sIgA play a critical role in maintaining intestinal environmental stability and intestinal autoimmune responses (Huang et al., 2020). In addition, SCFAs are closely linked to IgA production and can influence intestinal homeostasis by affecting IgA, and thus intestinal homeostasis. It has been revealed that administration of SCFAs mixture increases the expression of IgA or the levels of IgA secreted in various intestine regions, as well as the levels of IgA and immunoglobulin G (IgG) in the blood circulation (Kim et al., 2016). In AIH patients, the lack of SCFAs disrupts immune homeostasis by altering the levels of IgA, sIgA, and IgG, thereby contributing to the development of AIH. The absence of SCFAs in patients with AIH is a significant factor in the development of AIH.

#### 2.2.3 Disruption of immune cell homeostasis

Various immune cells interact and contribute to the maintenance of the body’s immune homeostasis. Changes in immune cells such as regulatory cells, Antigen-presenting (APC) cells, Th 1 cells, Th 2 cells, macrophages, Treg cells, and NKT cells have a significant effect on the immune system of the body. When such a system is dysfunctional, autoimmune diseases such as AIH as well as infections and tumors may develop. In addition to immune cell imbalances, the disruption of the systemic immune response that ultimately leads to AIH is also influenced by mucosal immunity (Wang et al., 2021). Metabolic disorders of gut microbes also contribute to the development of AIH by disrupting immune homeostasis via activation of immune cells or disruption of the dynamic equilibrium between immune cells (Cheng et al., 2022).

The imbalance of intestinal microflora will lead to inflammatory reactions and trigger self-reactive T cells (CD4^+^ T cells and CD8^+^ T cells), thus driving the occurrence of spontaneous autoimmune in target organs (Horai et al., 2015; Ruff et al., 2019). During AIH pathogenesis, APC cells activate T lymphocytes by mispresenting liver autoantigens as foreign antigens to uncharacterized T lymphocytes (Longhi et al., 2021). As unconventional APC cells, the presence of MHC class II molecules on the surface of hepatocytes has resulted in an enhanced autoimmune response to AIH (Gong et al., 2022). *In vitro* studies have shown that low concentrations of butyrate, a gut microbial metabolite, inhibit the proliferation of CD4^+^ T and CD8^+^ T cells (Corrêa-Oliveira et al., 2016). Many studies have shown that *B. fragilis*, a member of the intestinal microflora, can influence the differentiation and development of T lymphocytes by activating pattern recognition receptors (PRRs) such as Nucleotide-binding oligomerization domain 2 (NOD2), Toll-like receptors 1 (TLR1), Toll-like receptors 2 (TLR2) (Cheng et al., 2019). When stimulated by heat-killed *B. fragilis*, human peripheral blood mononuclear cells (PBMCs) have a greater capacity to produce interleukin-8 (IL-8) and interleukin-6 (IL-6) (Stappers et al., 2012). The dynamic balance of follicular regulatory T (TFR)/follicular helper T (TFH) cells also plays an important role in the immune homeostasis of the organism. Activated TFH cells participate in the autoimmune process and increase autoantibody secretion, whereas TFR cells inhibit the immune activation of TFH cells through the recognition of cytotoxic T-lymphocyte-associated protein 4 (CTLA4) while simultaneously inhibiting the development of autoimmunity in the organism. The elevated ratio of TFH cells induces pro-inflammatory factor production and aberrant activation of the immune system. Furthermore, the imbalance of the ratio of TFH cells to TFR cells plays an important role in the pathogenesis of the immunopathology of AIH (Liang et al., 2021). Activation of the TLR4/Myeloid differentiation factor 88 (TLR4/MyD88) signaling pathway by elevated LPS in the AIH model achieves both inhibition of TFR cells and activation of TFH cells (Levy et al., 2017). Inflammation and fibrosis of the liver caused by the immune system are closely linked to the activation of Kupffer cells by NKT cells and the recruitment of macrophages to secrete pro-inflammatory factors. When the intestinal barrier is compromised, intestinal microbiota and its metabolites, as intestinal antigens, pass through the barrier and transfer to the liver, activating hepatic dendritic cell (DC) *in situ* and subsequently causing NKT cell activation. When intestinal pathogens activate intestinal DC in the intestinal tract, intestinal DC is transmitted to the liver via Peyer patches, causing activation of hepatic NKT cells (Cheng et al., 2022).

Th cells, also referred to as T helper cells, engage in diverse subpopulations and interactions to activate macrophages and other immune cells for antigen phagocytosis and clearance. Th cells exhibit the ability to release a range of cytokines, contributing to various immune responses within the body. The balance between Th 1 and Th 2 cells is essential for the maintenance of cellular and humoral immunity, and an imbalance between the two disrupts immune homeostasis. On the surface of hepatocytes, Th 1 cells that secrete Interferon-gamma (IFN-γ) and interleukin-2 (IL-2), cytokines, and HLA class I antigen (HLA class I) and HLA class II are upregulated (Lobo-Yeo et al., 1990). Cytokines such as interleukin-4 (IL-4) and IL-10, which are secreted by Th 2 cells, enhance cytotoxicity by mediating the maturation of B cells into plasma cells that produce antibodies; if this cytotoxicity is not effectively inhibited, it is negatively correlated with the prognosis of the patients (Cochrane et al., 1976; Cochrane et al., 1978). Th 1 cell overactivation may trigger specific autoimmune diseases such as AIH, whereas Th 2 cell overactivation leads to the predisposition of the organism to infections and tumors. Additionally, it has been demonstrated that hepatocyte destruction mediated by Th 1 immune response plays a crucial role in the development of AIH (Yuksel et al., 2015). Notably, SCFAs inhibit the polarization process of Th 2 cells, causing a marked Th 1 tilt in the Th 1/Th 2 balance (Trompette et al., 2014). The acetylation of p70 S6 kinase and phosphorylation of rS6, which are essential for the mTOR pathway involved in the development of Th 17, Th 1, and IL-10^+^ T cells, were enhanced by inhibiting Histone Deacetylase (HDAC) in T cells using SCFAs (Park et al., 2015).

Th 17 and Treg cells are two subpopulations of lymphocytes with opposing functions, and the dynamic equilibrium of their ratios is crucial to the maintenance of the body’s normal immune response. The abnormal Th 17-cell response observed in AIH is associated with changes in the signaling of the AHR. These alterations lead to a diminished ability of Th 17 cells to respond to AHR activation. The increased proportion of Th 17 cells promotes the secretion of pro-inflammatory factors, such as tumor necrosis factor-α (TNF-α), which are intimately involved in the inflammatory injury of the liver and the activation of autoimmunity during its transitional phase. In contrast, Treg cells play an immunosuppressive function by secreting anti-inflammatory factors such as Transforming growth factor beta (TGF-β) and IL-10, which maintain a dynamic equilibrium with the ratio of Th 17 cells and regulate immune homeostasis under normal physiological conditions. Loss of immune tolerance is a key factor in the disruption of immune homeostasis in patients with AIH. Decreased numbers and functional defects of Treg cells, along with an increase in Th 17 cell polarization, may contribute to the loss of immune tolerance (Grant et al., 2014; Beringer and Miossec, 2018).

However, the alteration of intestinal microbiota leads to the destruction of the Treg/Th 17 immune balance, which causes an inflammatory response leads to the abnormal activation of immune response, and promotes the occurrence of AIH. In patients with AIH, the expression of Th 17 and Th 17-related cytokines (IL-17) was significantly elevated in the liver and peripheral blood, and the number of Th 17 cell infiltrates was strongly correlated with the severity of liver inflammation (Zhao et al., 2011; Beringer and Miossec, 2018). Even the ratio between Treg/Th 17 is related to the severity of AIH (Liu et al., 2021b). The imbalance between Treg and Th 17 cells may be caused by the interaction between intestinal parasites and the mucosal immune system (Khorasani et al., 2019). In addition, AHR activation can induce the upregulation of ectonucleoside triphosphate diphosphohydrolase 1 (CD39), an extracellular enzyme that hydrolyzes ATP to produce immunosuppressive adenosine, and the low level of CD39 is related to the imbalance between Treg and Th 17 cells (Vuerich et al., 2021). IL-17 also contributes to impaired Treg cell function, and increased amounts of IL-17 lead to polarization of newly generated Treg (ng Treg) toward a pro-inflammatory Treg phenotype with activated immune function (Longhi et al., 2012). It can induce immune cell infiltration and liver injury, which leads to liver inflammation and fibrosis. SCFAs, including butyrate, succinate, and propionate, have been suggested as small molecular mediators involved in microbial regulation of Treg cells. They exert their effects by inhibiting histone deacetylases, thereby modulating histone or FOXP3 acetylation, or by interacting with metabolite-sensing G-protein-coupled receptors, such as G-Protein Coupled Receptor 43(GPR43) or G protein-coupled receptor 109A (GPR109A) (Ramanan et al., 2023). Using IL-17 neutralizing antibodies can reduce serum alanine aminotransferase (ALT) levels and improve liver inflammation, which indicates that IL-17 plays an important regulatory role in the development of AIH (Zhao et al., 2011; Beringer and Miossec, 2018). Vuerich et al. found that elevated levels of HIF-1α in Th 17 cells and upregulation of aryl hydrocarbon receptor repressor (AHRR) and Estrogen Receptor-αPolypeptide (Erα) in Treg cells led to disruption of AHR signaling function and purinergic activity in Th 17 and Treg cells (Vuerich et al., 2021). Recent research has demonstrated that the proportion of Th 17-type and CD161-positive T cells, as well as the secretion of IL-22 by Th 22-type responses, is required for the normal epithelial barrier function (Walker et al., 2019; Alcorn, 2020). Extracellular Association of Tennis Professionals co-produced by gut microbiota leads to the activation of immune cells, which in turn leads to cell death. Besides, it also plays an important role in promoting the differentiation of Th 17 cells (Kayama et al., 2020). Polysaccharide A (PSA) in *B. fragilis* capsules is also involved in maintaining host immune balance. The inhibition of Th 17 cell polarization is also achieved by activating TLR in CD4^+^T cells and inhibiting Th 17-induced cytokine production (Cheng et al., 2019).

Receptor-interacting protein kinase-3 (RIP3), a crucial kinase involved in the signaling of necroptotic cell death, has been shown to have a role in autophagy. The kinase activity of RIP3 facilitates the recruitment of mixed lineage kinase domain-like (MLKL), leading to membrane permeabilization and the subsequent release of pro-inflammatory intracellular components (Zhang et al., 2023a). RIP3 signaling is related to the activation of macrophages/monocytes and the production of pro-inflammatory cytokines such as IL-6 (Zhang et al., 2018). IL-6, a key transforming factor, is implicated in the transformation of Treg into Th 17 cells that produce IL-17 and mediate the inflammatory process in the liver tissues of AIH patients. There is a correlation between its activity and the severity of liver injury. Taken together, RIP3-dependnt inflammation contributes to the development of AIH, suggesting its potential as a therapeutic target.

## 3 Conclusion

The intestinal microbiota, comprising over 10 billion microorganisms, is a intricate ecosystem within the human intestine, which maintains a dynamic balance with the host in a mutually beneficial symbiosis. It is involved in numerous physiological and pathological processes, including digestion and processing of food, maintenance of mucosal immune homeostasis, gastrointestinal inflammation, and the development of hepatic autoimmune diseases. Several physiological processes of the digestive system are carried out via bidirectional interactions between the gastrointestinal tract and the liver. 75% of the liver’s blood supply is conveyed by the portal vein from the intestines and spleen to the liver. Therefore, the liver’s physiology dictates that it is constantly exposed to intestinal microbiota and toxoid toxins, such as LPS and flagellin (Henao-Mejia et al., 2013). Thus, dynamic changes in the gut microbiota and its metabolites may have an impact on the normal physiologic function of the liver.

With advancing knowledge about the gut microbiota, an increasing number of studies are highlighting the potential significance of the gut microbiota in the development of liver diseases such as AIH, alcoholic liver disease (ALD), and nonalcoholic fatty liver disease (NAFLD). This may occur through mechanisms such as the disruption of intestinal barrier function and immune homeostasis (Cheng et al., 2022). However, only a small number of studies have evaluated the association between the autoimmune disease (AIH) and the intestinal microbiota. Due to the presence of numerous immune cells and innate immune receptors on the surface of various hepatocytes, intestinal microflora and its metabolites, dietary antigens, and so on, stimulate the immune response of the liver. Destruction of the intestinal barrier will result in liver damage, hepatic inflammation, and autoimmune diseases (Hsu and Schnabl, 2023). In genetically susceptible mice and patients with autoimmune diseases such as AIH, it has been demonstrated that the capacity of *Enterococcus gallinarum* to cross the intestinal barrier and transfer from the intestine to the liver leads to the development of autoimmune reactions (Manfredo Vieira et al., 2018). In AIH, the transfer of gut microbiota from the intestines to the liver is therefore the “initiator” of autoimmunity. Changes in the ratio of gut microbiota and metabolic disorders contribute to the pathogenesis of AIH by disrupting immune homeostasis and activating related signaling pathways. For example, the imbalance of immune cells, such as Treg/Th 17, Th 1/Th 2, TFR/TFH; the activation of immune cells, such as NKT cells; the increase in secretion of inflammatory factors, which disrupts the balance of immune homeostasis; the decrease in SCFAs and Tryptophan; and increased secretion of LPS, all of which damages the intestinal barrier and increases intestinal permeability through the activation of signaling pathways such as TLR, NF-kB, and AHR.

Clarifying the mechanism by which gut microbiota causes AIH is essential for the targeted search for effective therapeutic targets for AIH. The exploration of the gut microbial composition of AIH patients and animal models is conducted through the testing of their feces, which does not accurately reflect the true status of their gut microbes or track changes in the intestinal microbiota. In addition, due to the effects of medications, diet, and environment on the metabolism of gut microbiota, the changes in the gut microbiota of AIH patients, such as *Lactobacillus*, *Faecali bacterium*, and *Lachospiraceae* remain controversial in various studies (Cheng et al., 2022). In most studies, the use of 16S rRNA gene sequencing rather than metagenomics sequencing renders the sequencing results insufficient for functional analysis. More direct evidence and more precise assays are required to investigate the precise mechanisms by which the gut microbiota influences AIH, and larger patient populations and extended follow-up periods are essential.

## Author contributions

CS: Data curation, Writing – original draft. DZ: Data curation, Writing – original draft. QZ: Data curation, Writing – original draft. ZH: Data curation, Writing – original draft. YL: Data curation, Writing – original draft. DC: Conceptualization, Writing – review & editing.

## References

[B1] Airola C. Severino A. Porcari S. Fusco W. Mullish B. H. Gasbarrini A.. (2023). Future modulation of gut microbiota: from eubiotics to FMT, engineered bacteria, and phage therapy. Antibiotics 12, 868. doi: 10.3390/antibiotics12050868 37237771 PMC10215521

[B2] Albillos A. de Gottardi A. Rescigno M. (2020). The gut-liver axis in liver disease: Pathophysiological basis for therapy. J. Hepatol. 72, 558–577. doi: 10.1016/j.jhep.2019.10.003 31622696

[B3] Alcorn J. F. (2020). IL-22 plays a critical role in maintaining epithelial integrity during pulmonary infection. Front. In Immunol. 11, 1160. doi: 10.3389/fimmu.2020.01160 32582219 PMC7296169

[B4] Alizadeh A. Akbari P. Garssen J.. (2022). Epithelial integrity, junctional complexes, and biomarkers associated with intestinal functions. Tissue Barriers 10, 1996830. doi: 10.1080/21688370.2021.1996830 34719339 PMC9359365

[B5] Babu G. Mohanty B. (2023). Neurotensin modulation of lipopolysaccharide induced inflammation of gut-liver axis: Evaluation using neurotensin receptor agonist and antagonist. Neuropeptides 97, 102297. doi: 10.1016/j.npep.2022.102297 36368076

[B6] Beringer A. Miossec P. (2018). IL-17 and IL-17-producing cells and liver diseases, with focus on autoimmune liver diseases. Autoimmun. Rev. 17, 1176–1185. doi: 10.1016/j.autrev.2018.06.008 30321671

[B7] Biagioli M. Carino A. Fiorucci C. Marchianò S. Di Giorgio C. Roselli R.. (2019). GPBAR1 functions as gatekeeper for liver NKT cells and provides counterregulatory signals in mouse models of immune-mediated hepatitis. Cell. Mol. Gastroenterol. Hepatol. 8, 447–473. doi: 10.1016/j.jcmgh.2019.06.003 31226434 PMC6718949

[B8] Blaak E. E. Canfora E. E. Theis S. Frost G. Groen A. K. Mithieux G.. (2020). Short chain fatty acids in human gut and metabolic health. Beneficial Microbes 11, 411–455. doi: 10.3920/BM2020.0057 32865024

[B9] Blake S. J. Wolf Y. Boursi B. Lynn D. J. (2023). Role of the microbiota in response to and recovery from cancer therapy. Nat. Rev. Immunol. doi: 10.1038/s41577-023-00951-0 37932511

[B10] Bragazzi M. C. Venere R. Vignone A. Alvaro D. Cardinale V. (2023). Role of the gut&ndash;Liver axis in the pathobiology of cholangiopathies: basic and clinical evidence. Int. J. Mol. Sci. 24, 6660. doi: 10.3390/ijms24076660 37047635 PMC10095354

[B11] Brock K. E. Cooper R. L. (2023). The effects of doxapram blocking the response of gram-negative bacterial toxin (LPS) at glutamatergic synapses. Biology 12 (8), 1046. doi: 10.3390/biology12081046 37626932 PMC10451348

[B12] Bruneau A. Hundertmark J. Guillot A. Tacke F. (2021). Molecular and cellular mediators of the gut-liver axis in the progression of liver diseases. Front. Med. 8. doi: 10.3389/fmed.2021.725390 PMC8505679 34650994

[B13] Cheng H. Guan X. Chen D. Ma W. (2019). The th17/treg cell balance: A gut microbiota-modulated story. Microorganisms 7 (12). doi: 10.3390/microorganisms7120583 PMC6956175 31756956

[B14] Cheng Z. Yang L. Chu H. (2022). The gut microbiota: A novel player in autoimmune hepatitis. Front. In Cell. Infection Microbiol. 12, 947382. doi: 10.3389/fcimb.2022.947382 PMC9310656 35899041

[B15] Chopyk D. M. Grakoui A. (2020). Contribution of the intestinal microbiome and gut barrier to hepatic disorders. Gastroenterology 159, 849–863. doi: 10.1053/j.gastro.2020.04.077 32569766 PMC7502510

[B16] Cochrane A. M. Moussouros A. Smith A. Portmann B. Eddleston A. L. Williams R. (1978). Lymphocyte cytotoxicity in chronic active hepatitis: effect of therapy and correlations with clinical and histological changes. Gut 19, 308–314. doi: 10.1136/gut.19.4.308 648937 PMC1411920

[B17] Cochrane A. M. Moussouros A. Thomsom A. D. Eddleston A. L. Wiiliams R. (1976). Antibody-dependent cell-mediated (K cell) cytotoxicity against isolated hepatocytes in chronic active hepatitis. Lancet (London England) 1, 441–444. doi: 10.1016/S0140-6736(76)91472-0 55716

[B18] Corrêa-Oliveira R. Fachi J. L. Vieira A. Sato F. T. Vinolo M. A.R. (2016). Regulation of immune cell function by short-chain fatty acids. Clin. Trans. Immunol. 5, e73. doi: 10.1038/cti.2016.17 PMC4855267 27195116

[B19] Elsherbiny N. M. Rammadan M. Hassan E. A. Ali M. E. El-Rehim A. S.A. Abbas W. A.. (2020). Autoimmune hepatitis: shifts in gut microbiota and metabolic pathways among Egyptian patients. Microorganisms 8 (7). doi: 10.3390/microorganisms8071011 PMC7409351 32640728

[B20] Fan Y. Ying J. Ma H. Cui H. (2023). Microbiota-related metabolites fueling the understanding of ischemic heart disease. iMeta 2, e94. doi: 10.1002/imt2.94 PMC10989774 38868424

[B21] Floreani A. Restrepo-Jiménez P. Secchi M. F. Secchi M. F. De Martin S. Leung P. S.C.. (2018). Etiopathogenesis of autoimmune hepatitis. J. Autoimmun. 95, 133–143. doi: 10.1016/j.jaut.2018.10.020 30385083

[B22] Gao F. He Q. Wu S. Zhang K. Xu Z. Kang J.. (2023). Catalpol ameliorates LPS-induced inflammatory response by activating AMPK/mTOR signaling pathway in rat intestinal epithelial cells. Eur. J. Pharmacol. 960, 176125. doi: 10.1016/j.ejphar.2023.176125 37890606

[B23] Gong J. Tu W. Liu J. Tian D. (2022). Hepatocytes: A key role in liver inflammation. Front. In Immunol. 13, 1083780. doi: 10.3389/fimmu.2022.1083780 36741394 PMC9890163

[B24] Grant C. R. Liberal R. Holder B. S. Cardone J. Ma Y. Robson S. C.. (2014). Dysfunctional CD39(POS) regulatory T cells and aberrant control of T-helper type 17 cells in autoimmune hepatitis. Hepatol. (Baltimore Md.) 59, 1007–1015. doi: 10.1002/hep.26583 PMC6377365 23787765

[B25] Guo X. Okpara E. S. Hu W. Yan C. Wang Y. Liang Q.. (2022). Interactive relationships between intestinal flora and bile acids. Int. J. Mol. Sci. 23, 8343. doi: 10.3390/ijms23158343 35955473 PMC9368770

[B26] Gupta U. Dey P. (2023). Rise of the guardians: Gut microbial maneuvers in bacterial infections. Life Sci. 330, 121993. doi: 10.1016/j.lfs.2023.121993 37536616

[B27] Gutzeit C. Magri G. Cerutti A. (2014). Intestinal IgA production and its role in host-microbe interaction. Immunol. Rev. 260, 76–85. doi: 10.1111/imr.12189 24942683 PMC4174397

[B28] Hapfelmeier S. Lawson M. A. E. Slack E. Kirundi J. K. Stoel M. Heikenwalder M.. (2010). Reversible microbial colonization of germ-free mice reveals the dynamics of IgA immune responses. Sci. (New York N.Y.) 328, 1705–1709. doi: 10.1126/science.1188454 PMC3923373 20576892

[B29] Henao-Mejia J. Elinav E. Thaiss C. A. Licona-Limon P. Flavell R. A. (2013). Role of the intestinal microbiome in liver disease. J. Autoimmun. 46, 66–73. doi: 10.1016/j.jaut.2013.07.001 24075647

[B30] Horai R. Zárate-Bladés C. R. Dillenburg-Pilla P. Chen J. Kielczewski J. L. Silver P. B.. (2015). Microbiota-dependent activation of an autoreactive T cell receptor provokes autoimmunity in an immunologically privileged site. Immunity 43, 343–353. doi: 10.1016/j.immuni.2015.07.014 26287682 PMC4544742

[B31] Hov J. R. Karlsen T. H. (2023). The microbiota and the gut-liver axis in primary sclerosing cholangitis. Nature Reviews. Gastroenterol. Hepatol. 20 (3), 135–154. doi: 10.1038/s41575-022-00690-y 36352157

[B32] Hsu C. L. Schnabl B. (2023). The gut–liver axis and gut microbiota in health and liver disease. Nat. Rev. Microbiol. 21, 719–733. doi: 10.1038/s41579-023-00904-3 37316582 PMC10794111

[B33] Huang J. Pearson J. A. Peng J. Hu Y. Sha S. Xing Y.. (2020). Gut microbial metabolites alter IgA immunity in type 1 diabetes. JCI Insight 5 (10). doi: 10.1172/jci.insight.135718 PMC7259536 32298241

[B34] Iancu M. A. Profir M. Roşu O. A. Ionescu R. F. Cretoiu S. M. Gaspar B. S.. (2023). Revisiting the intestinal microbiome and its role in diarrhea and constipation. Microorganisms 11, 2177. doi: 10.3390/microorganisms11092177 37764021 PMC10538221

[B35] Jiang N. Xiao Y. Yuan K. Wang Z. (2023). Effect of intestinal microbiota on liver disease and its related future prospection: From the perspective of intestinal barrier damage and microbial metabolites. J. Gastroenterol. Hepatol. 38, 1056–1071. doi: 10.1111/jgh.16129 36662612

[B36] Kaetzel C. S. (2005). The polymeric immunoglobulin receptor: bridging innate and adaptive immune responses at mucosal surfaces. Immunol. Rev. 206, 83–99. doi: 10.1111/j.0105-2896.2005.00278.x 16048543

[B37] Kayama H. Okumura R. Takeda K. (2020). Interaction between the microbiota, epithelia, and immune cells in the intestine. Annu. Rev. Immunol. 38, 23–48. doi: 10.1146/annurev-immunol-070119-115104 32340570

[B38] Keir M. Yi T. Lu T. Ghilardi N. (2020). The role of IL-22 in intestinal health and disease. J. Exp. Med. 217, e20192195. doi: 10.1084/jem.20192195 32997932 PMC7062536

[B39] Khantakova J. N. Sennikov S. V. (2023). T-helper cells flexibility: the possibility of reprogramming T cells fate. Front. In Immunol. 14, 1284178. doi: 10.3389/fimmu.2023.1284178 38022605 PMC10646684

[B40] Kharrazian D. Herbert M. Lambert J. (2023). The relationships between intestinal permeability and target antibodies for a spectrum of autoimmune diseases. Int. J. Mol. Sci. 24 (22). doi: 10.3390/ijms242216352 PMC10671756 38003542

[B41] Khorasani S. Mahmoudi M. Kalantari M. R. Lavi Arab F. Esmaeili S.-A. Mardani F.. (2019). Amelioration of regulatory T cells by Lactobacillus delbrueckii and Lactobacillus rhamnosus in pristane-induced lupus mice model. J. Cell. Physiol. 234, 9778–9786. doi: 10.1002/jcp.27663 30370554

[B42] Kim M. Qie Y. Park J. Kim C. H. (2016). Gut microbial metabolites fuel host antibody responses. Cell Host Microbe 20, 202–214. doi: 10.1016/j.chom.2016.07.001 27476413 PMC4982788

[B43] Kronsten V. T. Tranah T. H. Pariante C. Shawcross D. L. (2022). Gut-derived systemic inflammation as a driver of depression in chronic liver disease. J. Hepatol. 76, 665–680. doi: 10.1016/j.jhep.2021.11.008 34800610

[B44] Levy M. Kolodziejczyk A. A. Thaiss C. A. Elinav E. (2017). Dysbiosis and the immune system. Nat. Rev. Immunol. 17, 219–232. doi: 10.1038/nri.2017.7 28260787

[B45] Li L. Kang Y. The gut microbiome and autoimmune hepatitis: implications for early diagnostic biomarkers and novel therapies. Mol. Nutr. Food Res. 67 (24), 2300043. doi: 10.1002/mnfr.202300043 37350378

[B46] Liang M. Liwen Z. Jianguo S. Juan D. Fei D. Yin Z.. (2021). Fecal microbiota transplantation controls progression of experimental autoimmune hepatitis in mice by modulating the TFR/TFH immune imbalance and intestinal microbiota composition. Front. In Immunol. 12, 728723. doi: 10.3389/fimmu.2021.728723 34912328 PMC8667314

[B47] Lin H. Lin J. Pan T. Li T. Jiang H. Fang Y.. (2023). Polymeric immunoglobulin receptor deficiency exacerbates autoimmune hepatitis by inducing intestinal dysbiosis and barrier dysfunction. Cell Death Dis. 14, 68. doi: 10.1038/s41419-023-05589-3 36709322 PMC9884241

[B48] Lin R. Zhou L. Zhang J. Wang B. (2015). Abnormal intestinal permeability and microbiota in patients with autoimmune hepatitis. Int. J. Clin. Exp. Pathol. 8, 5153–5160. 26191211 PMC4503083

[B49] Liu C. Wang Y.-L. Yang Y.-Y. Zhang N.-P. Niu C. Shen X.-Z.. (2021a). Novel approaches to intervene gut microbiota in the treatment of chronic liver diseases. FASEB J. 35, e21871. doi: 10.1096/fj.202100939R 34473374

[B50] Liu H. Zhao J. Zhang W. Nie C. (2022). Impacts of sodium butyrate on intestinal mucosal barrier and intestinal microbial community in a weaned piglet model. Front. In Microbiol. 13, 1041885. doi: 10.3389/fmicb.2022.1041885 PMC9879053 36713180

[B51] Liu Q. Tian H. Kang Y. Tian Y. Li L. Kang X.. (2021b). Probiotics alleviate autoimmune hepatitis in mice through modulation of gut microbiota and intestinal permeability. J. Nutr. Biochem. 98, 108863. doi: 10.1016/j.jnutbio.2021.108863 34517094

[B52] Liwinski T. Casar C. Ruehlemann M. C. Bang C. Sebode M. Hohenester S.. (2020). A disease-specific decline of the relative abundance of Bifidobacterium in patients with autoimmune hepatitis. Alimentary Pharmacol. Ther. 51, 1417–1428. doi: 10.1111/apt.15754 32383181

[B53] Lobo-Yeo A. Senaldi G. Portmann B. Mowat A. P. Mieli-Vergani G. Vergani D.. (1990). Class I and class II major histocompatibility complex antigen expression on hepatocytes: a study in children with liver disease. Hepatol. (Baltimore Md.) 12, 224–232. doi: 10.1002/hep.1840120208 2118117

[B54] Longhi M. S. Liberal R. Holder B. Robson S. C. Ma Y. Mieli-Vergani G.. (2012). Inhibition of interleukin-17 promotes differentiation of CD25- cells into stable T regulatory cells in patients with autoimmune hepatitis. Gastroenterology 142, 1526–1535.e1526. doi: 10.1053/j.gastro.2012.02.041 22387392

[B55] Longhi M. S. Mieli-Vergani G. Vergani D. (2021). Regulatory T cells in autoimmune hepatitis: an updated overview. J. Autoimmun. 119, 102619. doi: 10.1016/j.jaut.2021.102619 33652348 PMC8044040

[B56] Lou J. Jiang Y. Rao B. Li A. Ding S. Yan H.. (2020). Fecal microbiomes distinguish patients with autoimmune hepatitis from healthy individuals. Front. In Cell. Infection Microbiol. 10, 342. doi: 10.3389/fcimb.2020.00342 PMC7416601 32850468

[B57] Macori G. Fanning S. (2023). The next-generation tools for risk assessment and precision food safety in the One Health continuum. Eur. J. Public Health 33 (Supplement_2). doi: 10.1093/eurpub/ckad160.1032

[B58] Manfredo Vieira S. Hiltensperger M. Kumar V. Zegarra-Ruiz D. Dehner C. Khan N.. (2018). Translocation of a gut pathobiont drives autoimmunity in mice and humans. Sci. (New York N.Y.) 359, 1156–1161. doi: 10.1126/science.aar7201 PMC5959731 29590047

[B59] Manns M. P. Lohse A. W. Vergani D. (2015). Autoimmune hepatitis–update 2015. J. Hepatol. 62, S100–S111. doi: 10.1016/j.jhep.2015.03.005 25920079

[B60] Mar J. S. Ota N. Pokorzynski N. D. Peng Y. Jaochico A. Sangaraju D.. (2023). IL-22 alters gut microbiota composition and function to increase aryl hydrocarbon receptor activity in mice and humans. Microbiome 11, 47. doi: 10.1186/s40168-023-01486-1 36894983 PMC9997005

[B61] Muratori L. Lohse A. W. Lenzi M. (2023). Diagnosis and management of autoimmune hepatitis. BMJ (Clinical Res. ed.) 380, e070201. doi: 10.1136/bmj-2022-070201 36746473

[B62] Nakajima A. Vogelzang A. Maruya M. Miyajima M. Murata M. Son A.. (2018). IgA regulates the composition and metabolic function of gut microbiota by promoting symbiosis between bacteria. J. Exp. Med. 215, 2019–2034. doi: 10.1084/jem.20180427 30042191 PMC6080902

[B63] Papadakos S. P. Arvanitakis K. Stergiou I. E. Lekakis V. Davakis S. Christodoulou M.-I.. (2023). The role of TLR4 in the immunotherapy of hepatocellular carcinoma: can we teach an old dog new tricks? Cancers 15, 2795. doi: 10.3390/cancers15102795 37345131 PMC10216531

[B64] Park J. S. Gazzaniga F. S. Kasper D. L. Sharpe A. H. (2023). Microbiota-dependent regulation of costimulatory and coinhibitory pathways via innate immune sensors and implications for immunotherapy. Exp. Mol. Med. 55, 1913–1921. doi: 10.1038/s12276-023-01075-0 37696895 PMC10545783

[B65] Park J. Kim M. Kang S. G. Jannasch A. H. Cooper B. Patterson J.. (2015). Short-chain fatty acids induce both effector and regulatory T cells by suppression of histone deacetylases and regulation of the mTOR-S6K pathway. Mucosal Immunol. 8, 80–93. doi: 10.1038/mi.2014.44 24917457 PMC4263689

[B66] Ramanan D. Pratama A. Zhu Y. Venezia O. Sassone-Corsi M. Chowdhary K.. (2023). Regulatory T cells in the face of the intestinal microbiota. Nat. Rev. Immunol. 23, 749–762. doi: 10.1038/s41577-023-00890-w 37316560

[B67] Ríos-Covián D. Ruas-Madiedo P. Margolles A. Gueimonde M. de Los Reyes-Gavilán C. G. Salazar N. (2016). Intestinal short chain fatty acids and their link with diet and human health. Front. In Microbiol. 7, 185. doi: 10.3389/fmicb.2016.00185 PMC4756104 26925050

[B68] Ruff W. E. Dehner C. Kim W. J. Pagovich O. Aguiar C. L. Yu A. T.. (2019). Pathogenic autoreactive T and B cells cross-react with mimotopes expressed by a common human gut commensal to trigger autoimmunity. Cell Host Microbe 26 (1). doi: 10.1016/j.chom.2019.05.003 PMC8194364 31227334

[B69] Shen K. Din A. U. Sinha B. Zhou Y. Qian F. Shen B. (2023). Translational informatics for human microbiota: data resources, models and applications. Briefings Bioinf. 24 (3). doi: 10.1093/bib/bbad168 37141135

[B70] Stappers M. H. T. Janssen N. A. F. Oosting M. Plantinga T. S. Arvis P. Mouton J. W.. (2012). A role for TLR1, TLR2 and NOD2 in cytokine induction by Bacteroides fragilis. Cytokine 60, 861–869. doi: 10.1016/j.cyto.2012.08.019 22998942

[B71] Terziroli Beretta-Piccoli B. Mieli-Vergani G. Vergani D. (2022). HLA, gut microbiome and hepatic autoimmunity. Front. In Immunol. 13. doi: 10.3389/fimmu.2022.980768 PMC9433828 36059527

[B72] Trompette A. Gollwitzer E. S. Yadava K. Sichelstiel A. K. Sprenger N. Ngom-Bru C.. (2014). Gut microbiota metabolism of dietary fiber influences allergic airway disease and hematopoiesis. Nat. Med. 20, 159–166. doi: 10.1038/nm.3444 24390308

[B73] Tulkens J. Vergauwen G. Van Deun J. Geeurickx E. Dhondt B. Lippens L.. (2020). Increased levels of systemic LPS-positive bacterial extracellular vesicles in patients with intestinal barrier dysfunction. Gut 69, 191–193. doi: 10.1136/gutjnl-2018-317726 30518529 PMC6943244

[B74] Verma S. Reddy P. Sowdhamini R. (2023). Integrated approaches for the recognition of small molecule inhibitors for Toll-like receptor 4. Comput. Struct. Biotechnol. J. 21, 3680–3689. doi: 10.1016/j.csbj.2023.07.026 37576745 PMC10412839

[B75] Vuerich M. Harshe R. Frank L. A. Mukherjee S. Gromova B. Csizmadia E.. (2021). Altered aryl-hydrocarbon-receptor signalling affects regulatory and effector cell immunity in autoimmune hepatitis. J. Hepatol. 74, 48–57. doi: 10.1016/j.jhep.2020.06.044 32663496 PMC7749856

[B76] Walker E. M. Slisarenko N. Gerrets G. L. Kissinger P. J. Didier E. S. Kuroda M. J.. (2019). Inflammaging phenotype in rhesus macaques is associated with a decline in epithelial barrier-protective functions and increased pro-inflammatory function in CD161-expressing cells. GeroScience 41, 739–757. doi: 10.1007/s11357-019-00099-7 31713098 PMC6925095

[B77] Wang H. Wang G. Banerjee N. Liang Y. Du X. Boor P. J.. (2021). Aberrant gut microbiome contributes to intestinal oxidative stress, barrier dysfunction, inflammation and systemic autoimmune responses in MRL/lpr mice. Front. In Immunol. 12, 651191. doi: 10.3389/fimmu.2021.651191 33912174 PMC8071869

[B78] Wang J. Zhu N. Su X. Gao Y. Yang R. (2023). Gut-microbiota-derived metabolites maintain gut and systemic immune homeostasis. Cells 12, 793. doi: 10.3390/cells12050793 36899929 PMC10000530

[B79] Wei Y. Li Y. Yan L. Sun C. Miao Q. Wang Q.. (2020). Alterations of gut microbiome in autoimmune hepatitis. Gut 69, 569–577. doi: 10.1136/gutjnl-2018-317836 31201284

[B80] Wei Y. Zeng B. Chen J. Cui G. Lu C. Wu W.. (2016). Enterogenous bacterial glycolipids are required for the generation of natural killer T cells mediated liver injury. Sci. Rep. 6, 36365. doi: 10.1038/srep36365 27821872 PMC5099575

[B81] Weiner A. Turjeman S. Koren O. (2023). Gut microbes and host behavior: The forgotten members of the gut-microbiome. Neuropharmacology 227, 109453. doi: 10.1016/j.neuropharm.2023.109453 36738776

[B82] Xu Z. Jiang N. Xiao Y. Yuan K. Wang Z. (2022). The role of gut microbiota in liver regeneration. Front. In Immunol. 13, 1003376. doi: 10.3389/fimmu.2022.1003376 36389782 PMC9647006

[B83] Yan X. Li J. Wu D. (2023). The role of short-chain fatty acids in acute pancreatitis. Molecules 28, 4985. doi: 10.3390/molecules28134985 37446647 PMC10343743

[B84] Yang J. Yang S. Liao Y. Deng Y. Jiao Y. (2023). Histone deacetylase inhibitor butyrate inhibits the cellular immunity and increases the serum immunity of pearl oyster Pinctada fucata martensii. Fish Shellfish Immunol. 133, 108529. doi: 10.1016/j.fsi.2023.108529 36632915

[B85] Ye X. Li H. Anjum K. Zhong X. Miao S. Zheng G.. (2022). Dual role of indoles derived from intestinal microbiota on human health. Front. In Immunol. 13, 903526. doi: 10.3389/fimmu.2022.903526 35784338 PMC9248744

[B86] Yuksel M. Wang Y. Tai N. Peng J. Guo J. Beland K.. (2015). A novel “humanized mouse” model for autoimmune hepatitis and the association of gut microbiota with liver inflammation. Hepatol. (Baltimore Md.) 62, 1536–1550. doi: 10.1002/hep.27998 PMC4763614 26185095

[B87] Zhang J. Guo L. Liu M. Jing Y. Zhou S. Li H.. (2018). Receptor-interacting protein kinase 3 mediates macrophage/monocyte activation in autoimmune hepatitis and regulates interleukin-6 production. United Eur. Gastroenterol. J. 6, 719–728. doi: 10.1177/2050640618756124 PMC6068788 30083334

[B88] Zhang J. Han L. Ma Q. Wang X. Yu J. Xu Y.. (2023a). RIP3 impedes Mycobacterium tuberculosis survival and promotes p62-mediated autophagy. Int. Immunopharmacol. 115, 109696. doi: 10.1016/j.intimp.2023.109696 36638666

[B89] Zhang Y. Zhang D. Chen L. Zhou J. Ren B. Chen H.. (2023b). The progress of autoimmune hepatitis research and future challenges. Open Med. (Wars) 18, 20230823. doi: 10.1515/med-2023-0823 38025543 PMC10655690

[B90] Zhao L. Tang Y. You Z. Wang Q. Liang S. Han X.. (2011). Interleukin-17 contributes to the pathogenesis of autoimmune hepatitis through inducing hepatic interleukin-6 expression. PloS One 6, e18909. doi: 10.1371/journal.pone.0018909 21526159 PMC3079758

[B91] Zhu L. R. Li S. S. Zheng W. Q. Ni W. J. Cai M. Liu H. P. (2023). Targeted modulation of gut microbiota by traditional Chinese medicine and natural products for liver disease therapy. Front. In Immunol. 14, 1086078. doi: 10.3389/fimmu.2023.1086078 36817459 PMC9933143

